# Supplementary material for: Inter-annual and decadal changes in teleconnections drive continental-scale synchronization of tree reproduction
Source: Nat Commun. 2017 Dec 20;8:2205. doi: 10.1038/s41467-017-02348-9 (PMC5738406; doi:10.1038/s41467-017-02348-9)
Supplement: Supplementary file 1 — Supplementary information [file 41467_2017_2348_MOESM1_ESM.pdf]

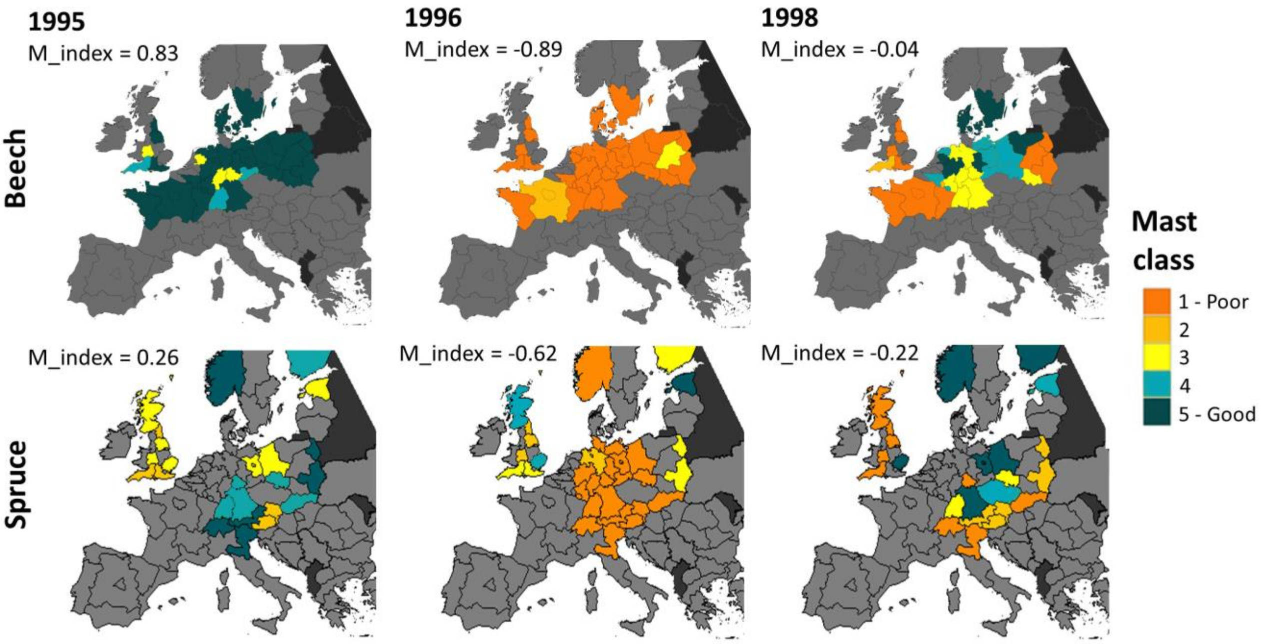

Supplementary Figure 1. Colored areas show the NUTS-1 used to build the masting index (M_index) at the sub-continental scale for beech (first raw) and spruce (second raw), respectively. Colors represent the modal masting class (class 1 – very poor: orange; class 5 – very abundant: blue) for a NUTS-1 in a given year. The three columns show a widespread full (1995), widespread failed (1996), and mixed (1998) masting and the corresponding beech and spruce M_index for the whole study area (M_index ranges from -1, indicative of widespread masting failure, and +1 when a widespread full mast occurs). Figure created using ggplot2 package for R[1].

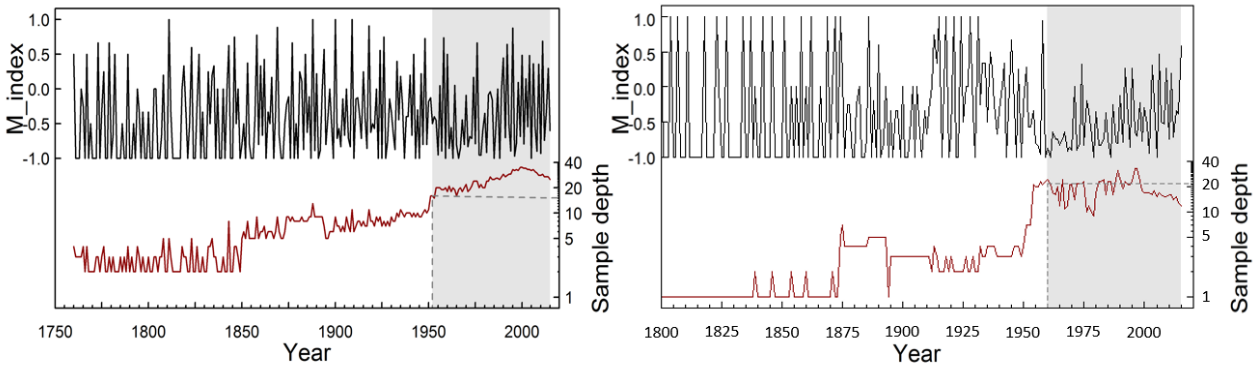

Supplementary Figure 2. Masting index (M-index) and sample depth (log scale of the number of NUTS-1 chronologies) used for computation for beech (left) and spruce (right). The grey shaded area shows the period used for model building when masting data are numerous and cover a large portion of the study area.

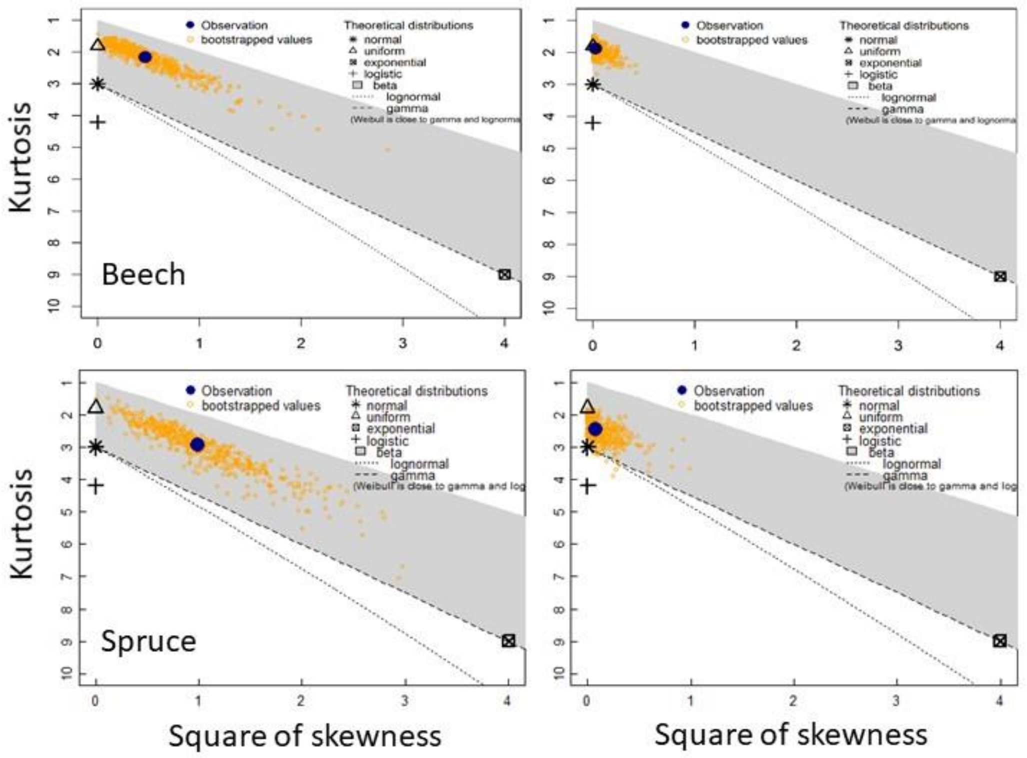

Supplementary Figure 3. Cullen and Frey graph created using the *descdist* from the fitdistrplus R package[2] to compare the skewness and kurtosis of the beech (first row) and spruce (second row) M_index distribution (blue dot) before (left column) and after (right column) the Freeman-Tukey[3] arcsine transformation. To account for uncertainty in the kurtosis and skewness estimated from the data, a nonparametric bootstrap procedure (n=500) was applied (orange dots). After transformation, M_index of both species approximates the skewness of a normal distribution (symbol *).

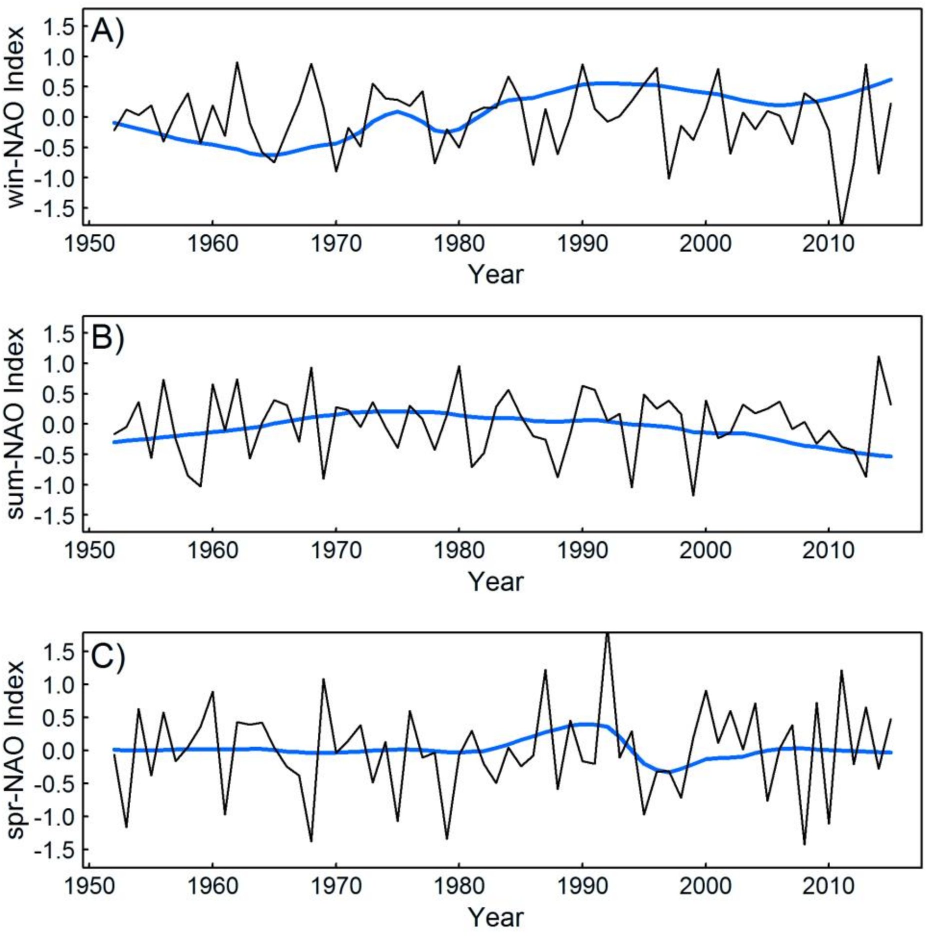

Supplementary Figure 4. High-frequency (black line) and low-frequency (blue line) components of seasonal NAO indices. A) Winter. B) Summer. C) Spring. This analysis uses the monthly NAO index provided by the Climate Prediction Centre – NOAA for the period 1950 to 2015. The low frequency component was fitted using the "*supsmu*" function of the *stats* R package in the frequency domain of 11 years (i.e. mean frequency domain identified by the wavelet coherence analysis between NAO and tree masting).

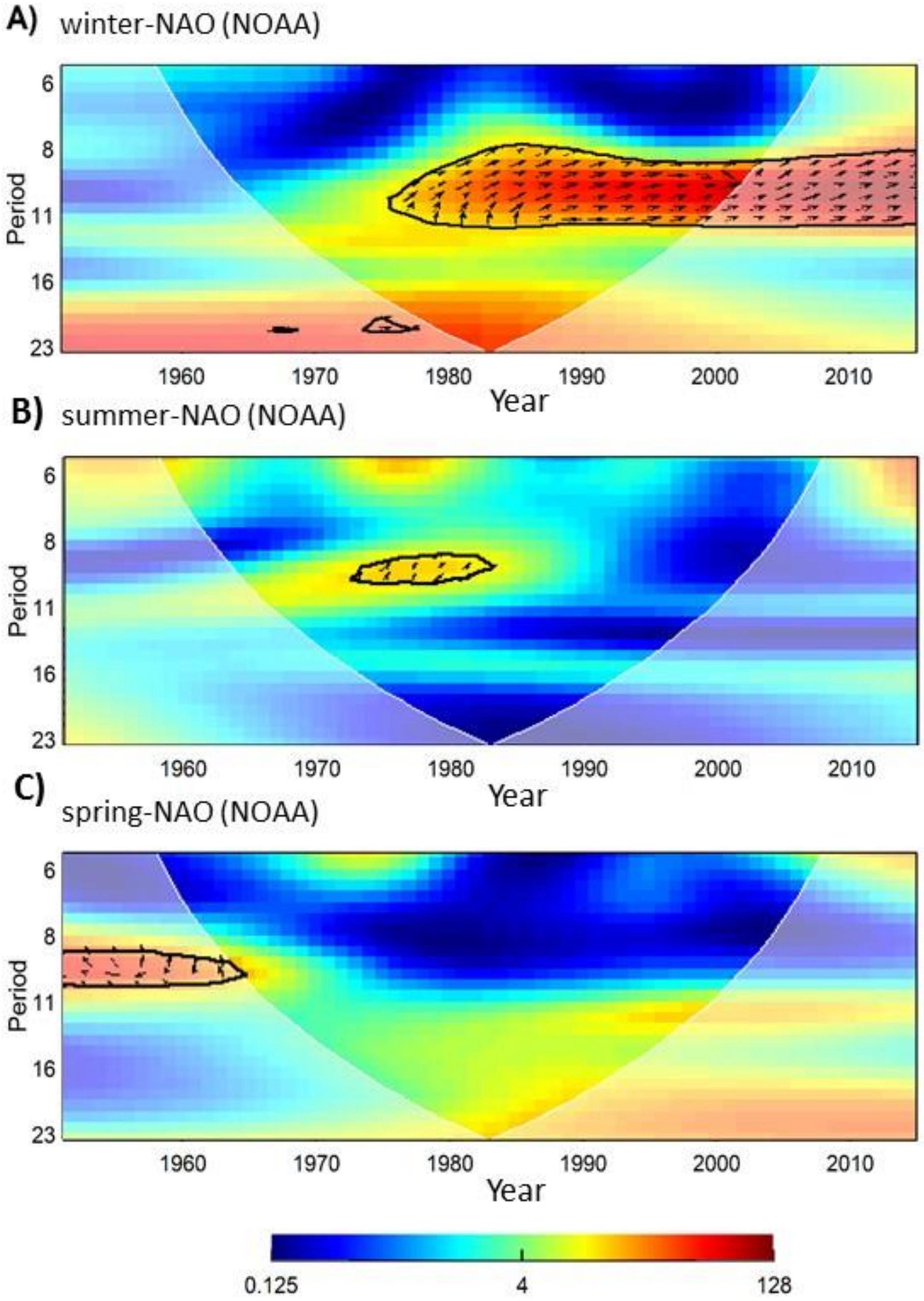

Supplementary Figure 5. Wavelet coherence between the standardized spruce M_index and indices for winter-NAO (A), summer-NAO (B) and spring-NAO (C). NAO indexes were computed using the Climate Prediction Centre-NOAA index. X-axes: years of analysis. Y-axes: frequency domain of the NAO-masting relationship in years. Arrows pointing right up-ward show in-phase behavior and y leading x, i.e. NAO leading M_index. Black contour designates frequencies of significant coherence (p<0.1, two-sided test); the white cone of influence shows the data space immune from distortion by edge effects.

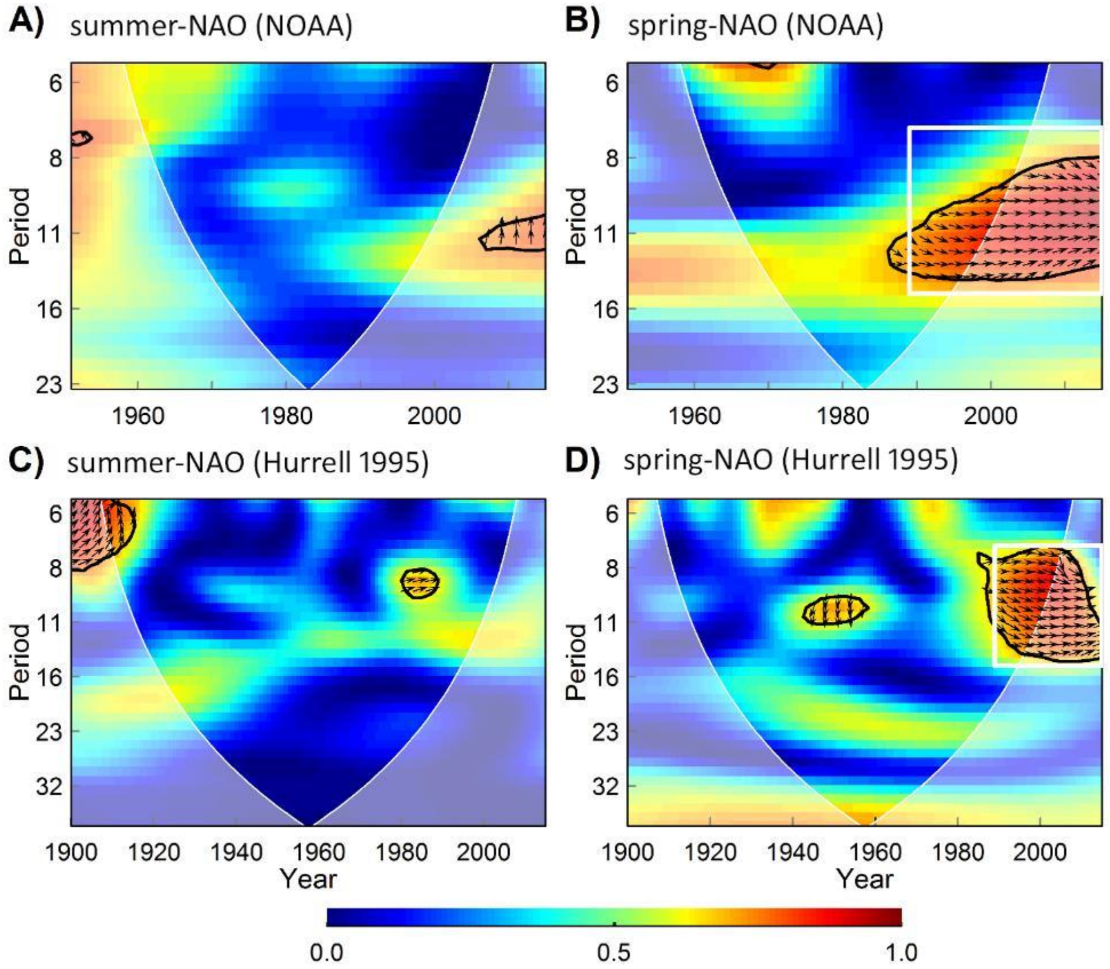

Supplementary Figure 6. Wavelet coherence between the standardized beech M_index and indices for summer-NAO (A and C) and spring-NAO (B and D). NAO indices are: Climate Prediction Centre-NOAA (A, B), Hurrell 1995 (C, D). X-axes: years of analysis. Y-axes: frequency domain of the NAO-masting relationship in years. Note that the x- and y-axes vary between plots. Arrows pointing right up-ward show in-phase behavior and y leading x, i.e. NAO leading M_index. Black contour designates frequencies of significant coherence ($p<0.1$, two-sided test); the white cone of influence shows the data space immune from distortion by edge effects. White boxes highlight areas of significant coherence between M_index and the NAO index. Note that boxes for B and D have the same dimension.

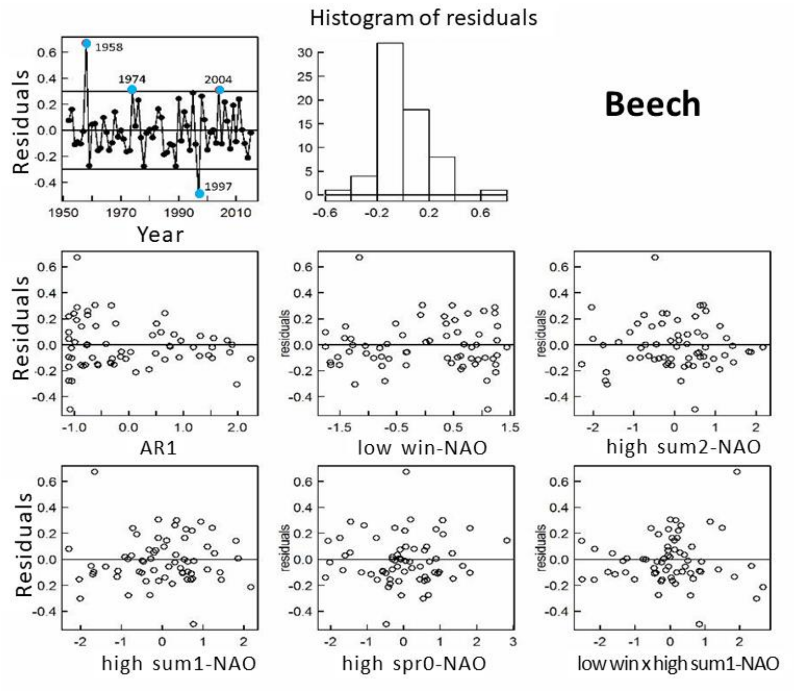

Supplementary Figure 7. Residuals of the final beech model including significant high- and low-frequency NAO components and the interaction term. Top row: chronology of residuals and histogram of residuals. Mid and bottom rows: residuals plotted against individual predictors included in the final model.

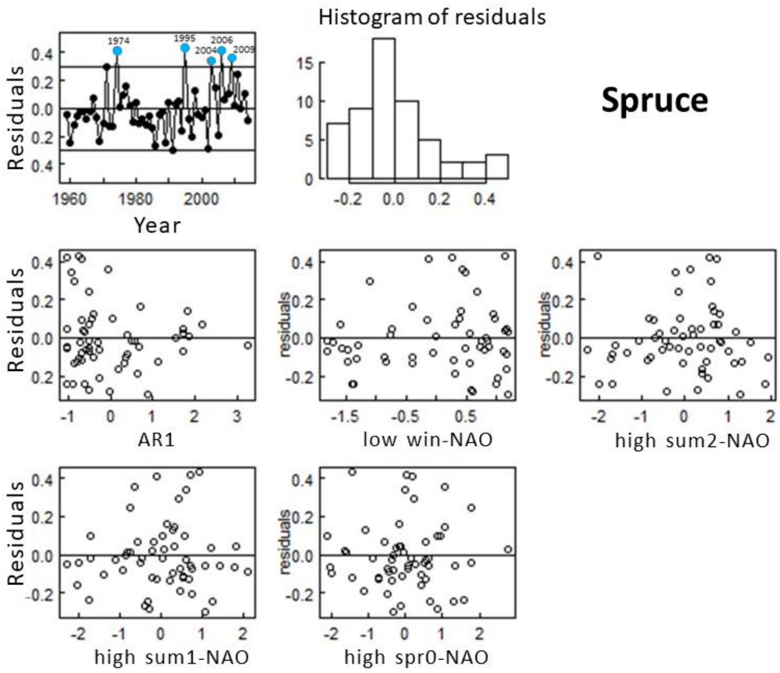

Supplementary Figure 8. Residuals of the final spruce model including significant high- and low-frequency NAO components and the interaction term. Top row: chronology of residuals and histogram of residuals. Mid and bottom rows: residuals plotted against individual predictors included in the final model.

Supplementary Table 1. Summary of the regression model predicting the interannual variability of large-scale beech (period 1952-2015) and spruce (period 1959-2014) masting in Central-Northern Europe testing as predictors raw seasonal NAO indexes by previous studies (Table 1). Standardized coefficients are shown as model estimates (β) ± standard error (SE). ΔAIC indicates the importance of the predictors and is calculated as the difference of AIC between the full model and the model without the predictor of interest.

**Species**	**European beech**				**Norway spruce**			
**Predictor**	**β**	**SE**	**p-value**	**ΔAIC**	**β**	**SE**	**p-value**	**ΔAIC**
AR1	-0.491	0.125	0.0001	-13.29	-0.137	0.128	0.3021	-0.92
summer-NAO $Y_{M\text{-}2}$	-0.571	0.117	0.0001	-19.32	-0.022	0.117	0.8515	1.96
winter-NAO $Y_{M\text{-}1}$	0.346	0.113	0.0022	-7.28	0.347	0.119	0.0037	-5.77
summer-NAO $Y_{M\text{-}1}$	0.326	0.114	0.0043	-5.48	0.120	0.131	0.362	1.14
spring-NAO Y_M	0.496	0.115	0.0001	-14.27	0.298	0.124	0.016	-3.72

Supplementary References

1. Wickham, H. ggplot2: Elegant Graphics for Data Analysis. Springer-Verlag New York (2009).
2. Freeman, M.F., Tukey, J.W. Transformations related to the angular and square root. *Ann. Math. Stat.*, **21**, 607-611 (1950).
3. Delignette-Muller, M.L., Dutang, C. Fitdistrplus: An R package for fitting distributions. *J. Stat. Softw.*, **64(4)**, 1-34 (2015).
