# Supplementary material for: Inter-annual and decadal changes in teleconnections drive continental-scale synchronization of tree reproduction
Source: Nat Commun. 2017 Dec 20;8:2205. doi: 10.1038/s41467-017-02348-9 (PMC5738406; doi:10.1038/s41467-017-02348-9)
Supplement: Supplementary file 3 — Description of Additional Supplementary Files [file 41467_2017_2348_MOESM3_ESM.pdf]

Description of Additional Supplementary Files

File Name: Supplementary Software
Description: The code is written R 3.3.1 language and contains all steps (data downloading and analysis) to reproduce statistical tests and figures.
